# Late lymphocele infection with *Parvimonas micra* in a kidney allograft recipient

**DOI:** 10.1186/s12879-025-10759-z

**Published:** 2025-03-28

**Authors:** Rihab Dkhissi, Gabriel Ouellet, Xavier Charmetant, Fanny Buron, Florent Valour, Olivier Rouviere, Xavier Matillon, Emmanuel Morelon

**Affiliations:** 1 Department of Nephrology, Dialysis and Transplantation, Ibn Sina Hospital, Rabat, Morocco; 2 CHU de Québec – Université Laval, Quebec City, Canada; 3 Department of Transplantation, Nephrology and Clinical Immunology, Edouard Herriot Hospital, Hospices Civils de Lyon, Lyon, 69003 France; 4 Department of Infectious and Tropical Diseases, Croix-Rousse Hospital, Hospices Civils de Lyon, Lyon, 69004 France; 5 Department of Medical and Interventional Radiology, Edouard Herriot Hospital, Hospices Civils de Lyon, Lyon, 69003 France; 6 Department of Urology and Transplant Surgery, Edouard Herriot Hospital, Hospices Civils de Lyon, Lyon, 69003 France

**Keywords:** Late lymphocele infection, *Parvimonas micra*, Kidney transplantation, Case report

## Abstract

**Background:**

Lymphocele infection is a frequent and usually early complication following renal transplantation. We report the case of a transplanted patient with a chronic lymphocele that became infected six years after transplantation *Parvimonas micra*, a commensal of the human oral cavity.

**Case presentation:**

The patient had a stable lymphocele for six years post-transplantation, as observed through several medical imaging studies, without the need for intervention due to the absence of any impact on graft function. Regarding a six-month progressive decline in general condition, a persistent inflammatory syndrome and a deterioration of renal function, a PET scan revealed a hypermetabolic infiltration behind the lymphocele adjacent to the graft. Bacterial superinfection with *Parvimonas micra* was diagnosed by an exploratory puncture. The patient had a history of dental periodontal treatments. The initial attempt at treatment with radiological drainage and three months of antibiotic therapy was unsuccessful. Faced with radiological deterioration despite treatment, the patient underwent surgical intervention for lavage with necessary antibiotic therapy for an additional six weeks. He achieved clinical remission, but metabolic activity persists within the site of a residual collection, and the patient remains closely observed.

**Conclusions:**

Infected lymphoceles should be considered in the differential diagnosis for patients presenting with nonspecific infectious and inflammatory symptoms, regardless of the time elapsed since renal transplantation. The treatment of this complication can be complex.

## Background

Lymphocele is a common complication after renal transplantation, typically appearing days to months after the surgery [1]. Its incidence varies from 0.6 to 33.9% [2, 3]. Although its etiopathogenesis remains uncertain, numerous recognized risk factors include extensive vascular dissection of the iliac axis, obesity [3], acute rejection, incomplete ligation of graft lymphatic vessels, high-dose corticosteroid therapy, cadaveric origin of the graft, recipient diabetes [4] and renal polycystic disease as the initial nephropathy [1, 5]. Lymphoceles can be asymptomatic or lead to complications linked to the compression of neighbouring structures. Infection can complicate up to 6% of post-transplant lymphoceles [6] within two months [7]. The following case reports a renal transplant patient with a chronicized lymphocele becoming infected six years after transplantation with Parvimonas micra (P. micra) in the context of dental periodontal treatments.

## Case presentation

The patient is a 43-year-old man receiving a kidney transplant after kidney failure caused by chronic interstitial nephritis secondary to nonsteroidal anti-inflammatory drug toxicity for treating ankylosing spondylitis. Past medical history only included grade 1 obesity and arterial hypertension. He underwent peritoneal dialysis for two years before transplantation without any infectious complications. He received an HLA-compatible kidney from an eighty-year-old brain-death deceased donor in 2016. The donor had satisfactory function with an eGFR of 80 ml/min and did not present any increased infectious risk. The donor was CMV-negative, EBV-positive, and Toxoplasmosis-positive; the other serologies were negative. There were no surgical particularities or immediate complications. Being non-immune, the patient received basiliximab induction therapy and maintenance immunosuppression with corticosteroids, mycophenolate mofetil, and tacrolimus. Graft function remained imperfect, with a nadir creatinine of 190 µmol/L expected, given the marginal donor confirmed on protocolar biopsies in the first year post-transplantation. The transplant was complicated by a large peri-graft and posterior lymphocele, measuring 89 × 86 × 170 mm, in the second week post-transplantation, on routine follow-up ultrasounds, without any indication for drainage. This collection remained stable over annual imaging follow-ups without intervention due to the absence of any impact on graft function or symptoms.

Six years post-transplant, in 2023, the patient was hospitalized for a six-month progressive insidious decline in general condition, characterized by a 10-kg weight loss, a persistent inflammatory syndrome with a C-reactive protein plateau around 30 mg/L, and a progressive kidney graft dysfunction reaching a creatinine level of 280 µmol/L. A PET scan revealed a hypermetabolic infiltration behind the posterior collection adjacent to the graft, extending 83 mm in contact with the psoas with hypermetabolism of the collection’s capsule of malignant appearance. Drainage removed 1500 ml of purulent fluid, with cytological analysis showing an inflammatory and necrotic appearance. Unexpectedly, bacteriological samples identified P. micra, a usual commensal of the human oral cavity. Mycological samples and the search for malignant cells were negative. The interrogation following bacterial identification highlighted frequent dental care sessions between 2021 and 2023, monthly for the 4 months before hospitalization. The infection occurred despite appropriate amoxicillin prophylaxis. The patient showed no other signs of distant superinfection, including no endocarditis. Following drainage, the biological inflammatory syndrome resolved, and graft function returned to baseline.

**Fig. 1:**
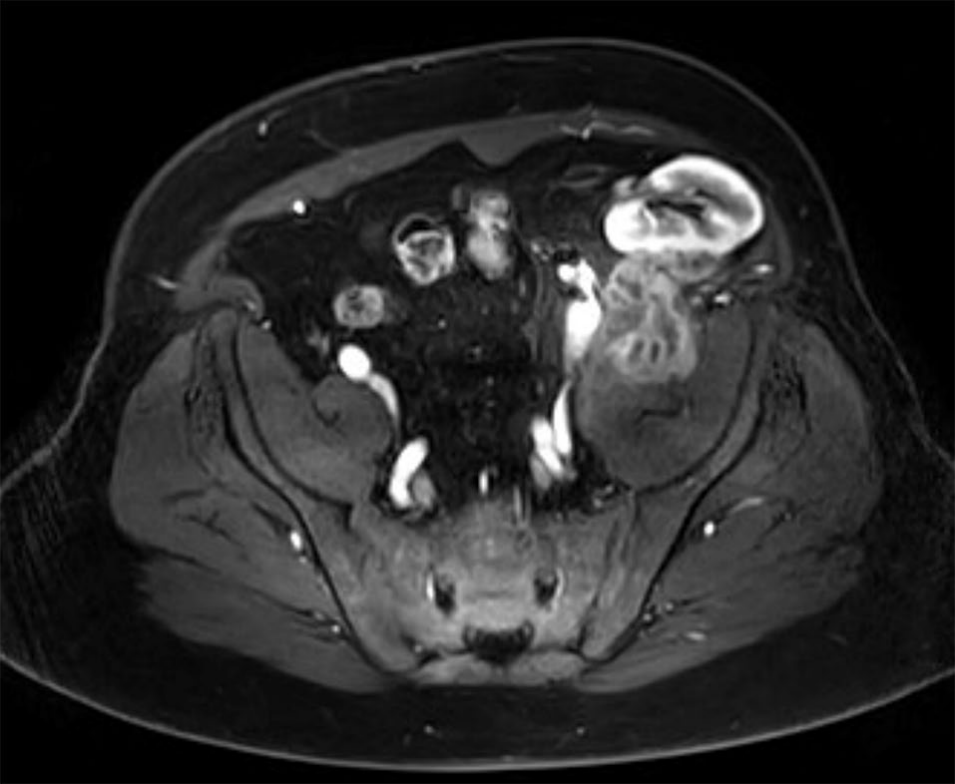
MRI of the collection following the first aspiration and drainage. A thick capsule is observed below the renal graft, compressing it on the posterior wall, initially raising suspicion of a tumoral nature

However, nine days post-drainage, considering the aspect of the partially solid collection, a re-evaluation using magnetic resonance imaging (MRI) revealed the persistence of a very thick capsule strongly enhancing, as well as the continued mass effect on the posterior part of the graft with an impression of parenchymal invasion suspicious of a malignant tumor (Fig. 1). An echo-guided biopsy confirmed the presence of P. micra, fibrosis and inflammatory cells and the absence of tumoral cells. The renal parenchyma was not sampled. The patient was started on his first antibiotic treatment for three months with 3 g of amoxicillin combined with 3 g of amoxicillin-clavulanate, which was chosen because of P. micra susceptibility, coverage of other oral cavity pathogens and distribution quality for a deep abscess. The triple immunosuppressive regimen remained unchanged considering minimal state and preserved general condition.

**Fig. 2:**
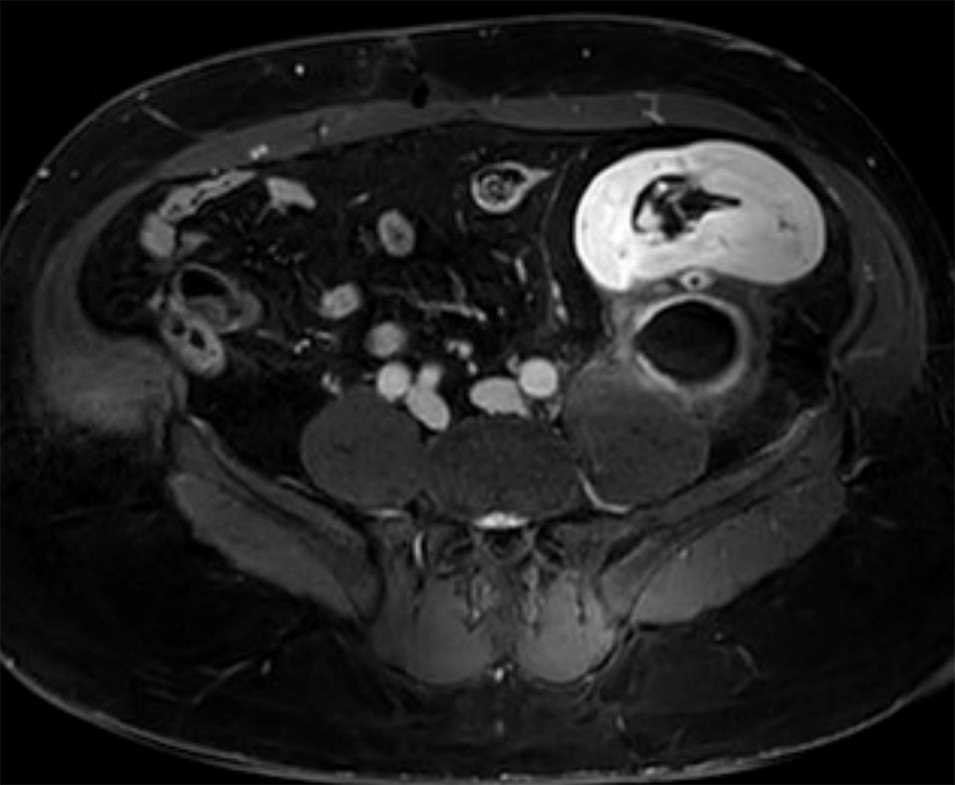
MRI showing the fluid collection with a thick and heterogeneous capsule before the second aspiration and drainage. The view is similar to Fig. 3, allowing comparison

**Fig. 3:**
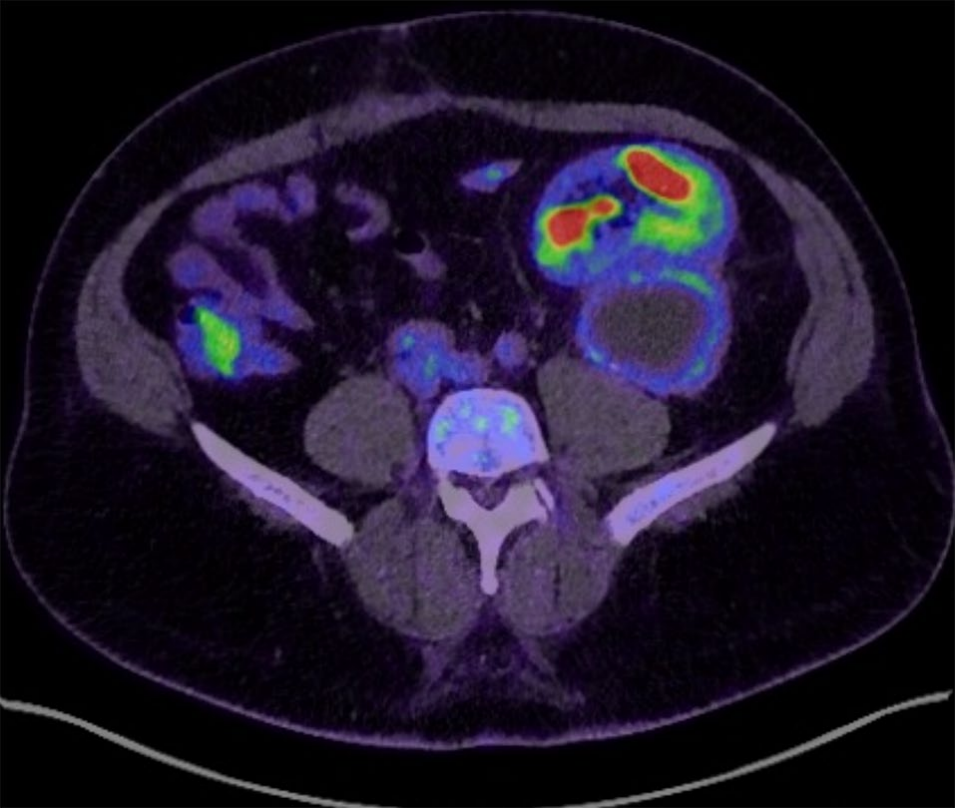
PET scan showing hypermetabolism of the lymphocele capsule despite the first drainage and antibiotic therapy

**Fig. 4:**
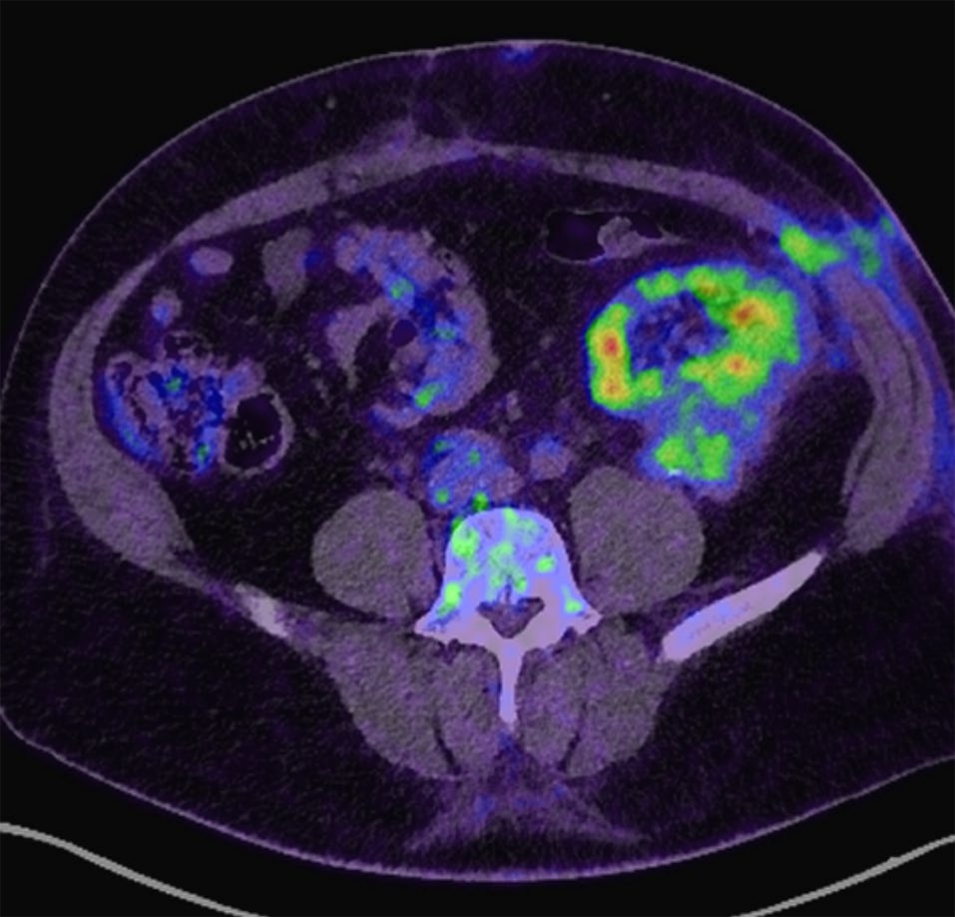
PET Scan showing nearly complete resolution of the liquid component of the collection with persistent metabolic activity in the still-present capsule

Despite adequate antibiotic therapy, a follow-up MRI after antibiotic completion showed the reconstitution of the liquid portion of the lymphocele and the persistence of an inflammatory peripheral capsule (Fig. 2). Clinically, the patient remained in good general condition, without any fever and biological inflammatory signs, despite hypermetabolism on the control PET scan (Fig. 3). A second echo-guided puncture was performed, extracting 200 ml of inflammatory fluid rich in polymorphonuclear cells. P. micra persisted in PCR analyses, but the culture remained sterile. A surgical lavage of the purulent collection via short laparotomy was performed, but unfortunately, it was incomplete due to adherence. The antibiotic therapy (amoxicillin and clavulanate) was therefore continued. Bacteriological samples (two deep tissue and four intra-abdominal fluid culture samples) revealed the eradication of P. micra and the presence of Staphylococcus epidermidis in one of the samples. However, sequencing through a metagenomic approach of amplified bacterial DNA has revealed the presence of P. micra. The patient was placed on clindamycin at 2400 mg daily to address sensitive bacteria. Ultrasound evaluation three weeks after surgical intervention and antibiotic therapy found a 24-mm residual collection near the upper pole of the transplant. Renal function remained stable at baseline. Clindamycin therapy was continued for three additional weeks. After 6 weeks of antibiotics, the control PET scan showed nearly complete resolution of the liquid component of the collection with persistent metabolic activity in the still-present capsule (Fig. 4). No sign of infection relapse has been noted in a year close follow-up. The patient remains under close clinical and biological monitoring.

## Discussion and conclusions

*Lymphocele infection* is a complication that typically occurs early after pelvic surgery, ranging from a few days to months after renal transplantation [7], ten months following renal graft removal and up to 10 months after urological surgery [8]. Only two cases of delayed superinfection of lymphoceles have been reported after more than a year of urological surgery [9, 10]. Hematogenous spread is possible [8, 9, 11] and Gram-positive cocci organisms cause it, particularly Staphylococcus aureus in two cases in the literature after kidney transplantation [7].

Our case is unique as it illustrates the possibility, considering the absence of a positive blood culture for the same germ and the presumption that the source of the bacteria is dental care, of late hematogenous infection of a lymphocele occurring six years after renal transplantation. A thorough review of the literature found no similar cases described.

Parvimonas micra, formerly known as Peptostreptococcus micros or Micromonas micros [12], is a Gram-positive anaerobic commensal of the subgingival dental plaque, causing periodontal problems such as periodontitis [13], particularly in patients with kidney failure or under immunosuppression after renal transplantation [14]. Due to immunosuppression, P. micra can become more invasive and be responsible for intra-abdominal abscesses [11, 14, 15].

The treatment of infected lymphoceles consists of appropriate antibiotic therapy with or without lymphocele drainage [7, 9]. However, P. micra is more challenging to eradicate and requires a longer duration of antibiotic therapy [15].

This case report supports that superinfection of a lymphocele should be considered in cases of an unclear inflammatory syndrome, even years after renal transplantation, especially if the morphological or metabolic aspect of imaging changes over time.

## Data Availability

No datasets were generated or analysed during the current study.

## Abbreviations

BMI: Body mass index
MRI: Magnetic resonance imaging
P. micra: Parvimonas micra
PCR: Polymerase chain reaction
PET scan: Positron emission tomography
